# Prevalence, pattern and predictors of self-medication for COVID-19 among residents in Umuahia, Abia State, Southeast Nigeria: policy and public health implications

**DOI:** 10.1186/s40545-022-00429-9

**Published:** 2022-05-02

**Authors:** Chidinma Ihuoma Amuzie, Kalu Ulu Kalu, Michael Izuka, Uche Ngozi Nwamoh, Uloaku Emma-Ukaegbu, Franklin Odini, Kingsley Metu, Chigozie Ozurumba, Ijeoma Nkem Okedo-Alex

**Affiliations:** 1 Department of Community Medicine, Federal Medical Centre, Umuahia, Abia Nigeria; 2 Nigeria Field Epidemiology and Laboratory Training Program, Abuja, Nigeria; 3 Department of Community Medicine, Alex Ekwueme Federal University Teaching Hospital, Abakaliki, Ebonyi Nigeria; 4 African Institute for Health Policy and Health Systems, Ebonyi State University, Abakaliki, Ebonyi Nigeria

**Keywords:** COVID-19, Self-medication, Prevalence, Pattern, Pandemic

## Abstract

**Background:**

COVID-19 has led to restrictions on movements and lockdown measures, which have resulted to higher utilization of over-the-counter drugs compared to prescription-only drugs. This study determined the prevalence, pattern and predictors of self-medication for COVID-19 prevention and treatment.

**Methods:**

A cross-sectional survey was conducted between October and November 2021 among the residents of Umuahia, Abia State. The respondents were selected using a snowball sampling technique, and a self-administered semi-structured questionnaire was used to collect data on the variables via Google forms. Descriptive, bivariate and multivariate analyses were done using IBM SPSS version 26. The level of significance was set at 5%.

**Results:**

A total of 469 respondents participated in the survey. The overall prevalence of self-medication for COVID-19 prevention and treatment was 30.3% (95%CI: 26.7–34.1). The most commonly used medication was herbal products (43.7%). This was mainly self-prepared (41.5%). The major source of information for self-medication was from family members (39.4%). The majority of the respondents reported fear of isolation (76.3%), followed by fear of stigmatization (75.7%) as the triggers of self-medication. Older age (aOR = 1.87, 95% CI: 1.11–3.13), lower educational status [No formal education (aOR = 3.78, 95% CI: 1.28–11.19)], [Primary education (aOR = 2.15, 95% CI: 1.17–3.097)] and perception to cost (aOR = 2.29; 95CI: I.24–4.24) were the predictors of self-medication.

**Conclusion:**

Every one in three residents of Umuahia, Abia State, practiced self-medication for COVID-19 prevention and treatment. Some economic and socio-demographic factors were significantly associated with self-medication. We recommend intensifying public awareness campaigns on the risk of self-medication.

**Supplementary Information:**

The online version contains supplementary material available at 10.1186/s40545-022-00429-9.

## Background

The World Health Organization (WHO) declared COVID-19, a newly emerging disease of public health emergency of international concern on 30th January 2020 [1]. The COVID-19 pandemic has resulted in high morbidity and mortality globally. As of 12th January 2022, it has infected over 300 million people, with the resultant deaths of approximately 5.6 million people globally. As of the same reference date, over 200,000 cases and about 3086 deaths have been recorded in Nigeria [2]. Presently, Nigeria is in the third wave of the pandemic. Vaccines have been delivered to the Country (Nigeria) through the COVAX mechanism. However, uptake is low among the population. In Nigeria, a study noted a vaccine hesitancy rate of 41.8% in the general populace [3].

According to WHO, self-medication has been defined as the selection and use of medicine by individuals to treat self-recognized symptoms or illnesses [4]. This novel disease has led to restrictions on movement and lockdown measures. This has accounted for the higher utilization of over-the-counter (OTC) drugs compared to prescription-only medications (POMs) [5]. It has also been documented that changes made in the utilization of medical services during this pandemic are influencing self-medication [6]. Other illnesses seem to be neglected due to the burden of COVID-19 on the health system and people perceive healthcare workers as carriers of the virus, hence they keep away from them [7, 8]. Infodemics, lack of trust and confidence in the government regarding the safety of vaccines, contribute to vaccine hesitancy [9–11], leading to more procurement of self-medication.

Self-medication is known to exhibit both positive and negative outcomes [5]. The term which best describes the positive effects refers to responsible self-medication, as against inappropriate self-medication with the associated burden on the ecosystem [4]. There has been a rise in the sales of OTC drugs and controlled drugs in the era of the COVID-19 pandemic [12]. This has led to an increase in the burden of self-medication. In Nigeria, some of the OTC drugs include vitamins, anti-anaemia medicines, antacids, antimalarials, analgesics and cold relief [13]. Most drugs abused with no clinical evidence of COVID-19 treatment include hydroxychloroquine, azithromycin, ivermectin, calcium supplements and some antiretrovirals [14]. Additionally, traditional herbs are not left out of the scenario [15]. Herbal medicines, according to WHO, refer to “herbs, herbal materials, herbal preparations and finished herbal products, that contain whole plants, parts of plants, or other plant materials, including leaves, bark, berries, flowers, and roots, and/or their extracts as active ingredients intended for human therapeutic use or for other benefits in humans” [16]. In Africa, they are used as alternative medicines and play a crucial role in the treatment of diseases [17].

A study in Kenya noted an increase in self-medication from 36.2% pre-pandemic to 60.2% amid the pandemic [18]. Self-medication works against the non-pharmaceutical measures of COVID-19, giving a false sense of protection against the infection [15]. The toxicity of drugs used for self-medication of COVID-19 has been documented [7, 19, 20]. In Nigeria, deaths were recorded among people who self-medicated with chloroquine for the prevention and treatment of COVID [21]. Self-medication is known as one of the triggers for antimicrobial resistance (AMR) [22, 23]. Some infections have been left untreatable with the existing antimicrobials due to the upsurge of AMR [24]. A rising trend of economic burden, which includes the excess cost of treatment for infections caused by AMR for patients, has been reported [25]. It is known that self-medication predisposes one to drug overdose, hazardous drug reactions and drug dependence [26].

Various motivations for self-medication have been reported, including previous disease experience, perceived cues to action, high cost of medical services, existing bureaucracy in health facilities, and weak regulatory activities [14, 27]. Self-medication for COVID-19 is of public health importance, and there is a need to understand the practice and triggers of self-medication in the population. Results from this study will provide the information needed to design awareness programmes and formulate policies to tackle the mayhem of self-medication for COVID-19 prevention and treatment. This study aimed to determine the prevalence, pattern and predictors of COVID-19 prevention and treatment.

## Methods

### Study design and setting

This was a descriptive cross-sectional study conducted in Umuahia, Abia State. Umuahia is the capital of Abia state, one of the five states in the South-East zone of Nigeria. It has an estimated population of 303,787 in 2018 projected from the 2006 national population census with an annual growth rate of 2.7% [28]. It has 517 public primary healthcare centres, 17 public secondary healthcare facilities, and two public tertiary healthcare centres. There are many wholesale and retail pharmacies distributed in Umuahia. Many outlets of patent medicine stores are also visible within and around the main city.

### Study population

Adult residents in Umuahia made up the study population. The inclusion criteria included those aged ≥ 18 years and living in Umuahia for the past 3 months prior to the study period. Eligible participants who had chronic or debilitating illnesses interfering with communication were excluded from the study. A chronic or debilitating illness “is a physical or mental health condition that lasts more than one year and causes functional restrictions or requires ongoing monitoring or treatment” [29].

### Sample size determination

Using the proportion of 41% of self-medication for COVID-19 prevention and treatment in a previous study conducted in Nigeria[30] and precision of 5%, the sample size was calculated using the single proportion population formula. It is given as *n* = (*Z*_A_)^2^*pq*/*d*^2^. The minimum sample size was estimated to be 414. A non-response rate of 10% was assumed.

### Study tool and data collection process

An extensive literature review was performed and the structure of the questionnaire was developed based on published research. Using Google Forms, which is a cloud-based survey tool powered by Google, a structured questionnaire was created for data collection over a month (October 2021). The questionnaire was validated using the face and content validity techniques. The introductory section emphasized the privacy of the respondents’ responses. The pretest was done in a community outside the study area. This helped to improve the diction of the questionnaire. The questionnaire has different sections. Section 1 includes information on sociodemographics such as age, sex, marital status, religion, denomination, occupation status and income. Section 2 addresses knowledge of self-medication. Section 3 focuses on the prevalence of self-medication. Section 4 contains information on self-medication practices and Sect. 5 addresses the triggers of self-medication. The questionnaire link was distributed to various social media platforms, including Telegram, Facebook Messenger and WhatsApp Messenger_._ This was done through the contact lists of the researchers, in addition to neighbours, friends and relatives on different fora. They were encouraged to further share the link with their relatives, friends, colleagues and other broader social network. On a daily basis, a crafted reminder message containing the link to the questionnaire was sent out to the targeted WhatsApp, Facebook messenger and Telegram groups.

### Measurement of variables

The dependent variable was self-medication for COVID-19 prevention and treatment. It was coded as binary responses, ‘yes’ as ‘1’ and ‘no’ as ‘0’. The independent variables included the sociodemographics, self-medication practices and triggers of self-medication.

### Statistical analysis

Data coding, entry, cleaning, and analysis were done using IBM SPSS version 26 statistical program for Windows. Descriptive statistics was used to characterize the sample and study variables. Associations between independent variables and self-medication for COVID-19 prevention and treatment were assessed with cross-tabulations, followed by multivariate analysis using logistic regression to determine the significant independent predictors of self-medication for COVID-19 prevention and treatment. The level of significance was predetermined at a p-value of less than 0.05.

## Results

A total of 469 respondents participated in the survey. The mean age was 39.9 ± 13.5 years. The less than 30 years age group 129 (27.5%) and 31–40 years age group 128 (27.3%) were similar in proportion. Females 267 (56.9%) were more prevalent compared to males. Among the respondents, 203 (43.3%) had attained tertiary education and 288 (61.4%) were married. The majority of the respondents were Christians 460 (98.1%) and 219 (46.7%) of them belonged to the orthodox denomination. A greater proportion of the respondents were salary earners 199 (42.4%) with an equal proportion of the respondents 140 (29.9%) each belonging to the income categories of < 50,000 ($121) and > 100,000 ($242) (Table 1).

**Table 1:** Socio-demographic characteristics of respondents (*N* = 469)

Variables	Frequency	Percentage (%)
*Age*		
≤ 30	129	27.5
31–40	128	27.3
41–50	118	25.2
51–60	55	11.7
> 60	39	8.3
Mean	39.9 ± 13.5 years	
*Sex*		
Male	202	43.1
Female	267	56.9
*Educational status*		
None/primary	20	4.3
Secondary	115	24.5
Tertiary	203	43.3
Post graduate	131	27.9
*Marital status*		
Single	135	28.8
Married	288	61.4
Cohabiting	12	2.6
Widowed	27	5.8
Divorced	7	1.5
*Religion*		
Christianity	460	98.1
Others	9	1.9
*Denomination*		
Catholic	84	17.9
Orthodox	219	46.7
Pentecostal	152	32.4
*Employment status*		
Salary earner	199	42.4
Self employed	148	31.6
Unemployed	122	26.0
*Monthly household income (Naira)*		
No income	110	23.5
< 50,000	140	29.9
50,000–100,000	79	16.8
> 100,000	140	29.9

Almost all respondents 458 (97.7%) were aware of self-medication. More than three-quarters of the respondents 405 (88.4%) gave the correct definition of self-medication and majority of them 394 (86.0%) believed that self-medication was harmful. The prevalence of self-medication for the prevention and treatment of COVID-19 was 30.3% (95% CI: 26.7–34.1).

**Fig. 1:**
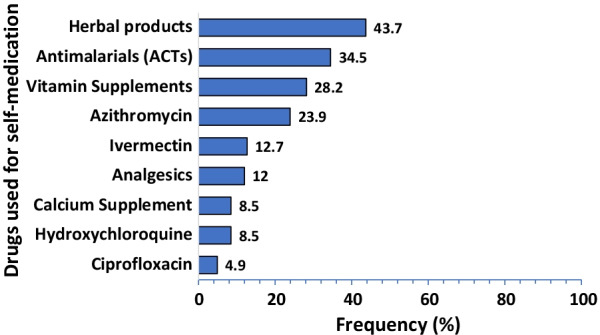
Drugs and supplements used for self-medication among the respondents (*n* = 142)

Figure 1 shows that most of the drugs used for self-medication in the prevention or treatment of COVID-19 were herbal products (43.7%) and antimalarials-ACTs (34.5%). This was followed by vitamin supplements (28.5%). Ciprofloxacin was the least mentioned drug used for self-medication against COVID-19 prevention and treatment.

**Fig. 2:**
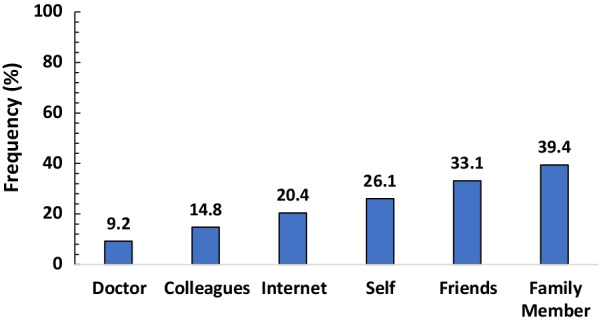
Source of information for self-medication among the respondents (*n* = 142)

Figure 2 reveals that the most prevalent source of information for self-medication was from family members (39.4%), followed by friends (33.1%). The internet as a source of information was mentioned by only 20.4% of the respondents.

**Fig. 3:**
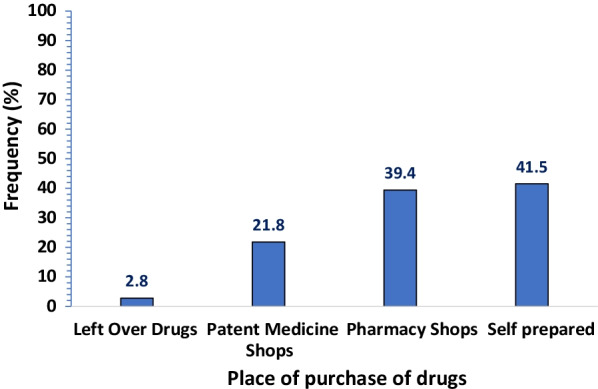
Place of purchase of drugs used for self-medication (*n* = 142)

As shown in Fig. 3, the majority of the respondents prepared the products used for self-medication by themselves, while 39.4% of the respondents got the drug products from pharmacy stores. A minority of the respondents (2.8%) used leftover drugs for self-medication against COVID-19 prevention and treatment.

**Fig. 4:**
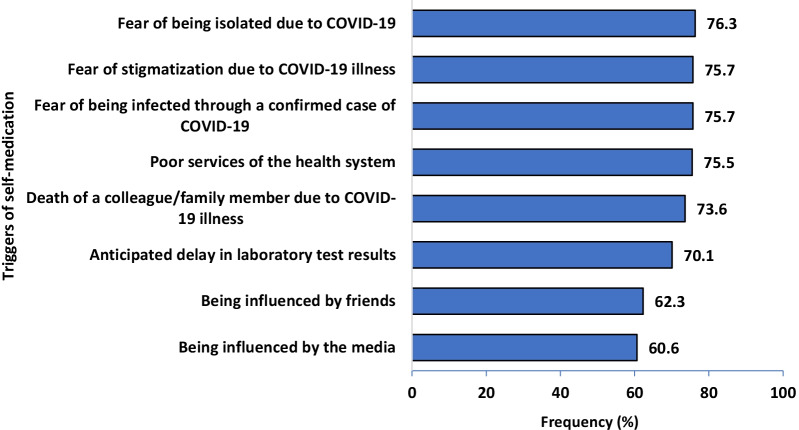
Triggers of self-medication among the respondents (*N* = 469)

Figure 4 shows the list of potential triggers for self-medication among the respondents. A majority of the respondents believed that self-medication for COVID-19 treatment and prevention could be triggered by fear of being isolated due to COVID-19 (76.3%), fear of stigmatization and fear of being infected by a confirmed case of COVID-19 (75.7%). Being influenced by friends (62.3%) and the media (60.6%) were the least mentioned by the respondents as triggers for self-medication.

In the bivariate logistic regression model, age, educational status, marital status and perception of the cost of self-medication were significantly associated with the practice of self-medication in the prevention and treatment of COVID-19. Those aged over 40 were 80% more likely to practice self-medication compared to those younger than 40 years (OR = 1.80; 95% CI:1.20–2.68). Those with no formal or primary education were more likely to self-medicate compared to the postgraduate degree holders (OR = 5.51; 95% CI:2.05–14.81). Similarly, those who had attained secondary education were also more likely to self-medicate compared to the postgraduates (OR = 2.36; 95% CI:1.34–4.14). Respondents who were widowed or separated were threefold more likely to self-medicate compared to singles (OR = 2.98; 95% CI: 1.36–6.53). Those who perceived that self-medication was cheaper were more likely to self-medicate compared to their counterparts (OR = 2.46; 95%CI: 1.40–4.33) (Table 2).

**Table 2:** Factors associated with self-medication for COVID-19 prevention and treatment (*N* = 469)

Variables	Self-medication		cOR	95% CI	*P* value*
	Yes *n *(%)	No *n *(%)			
*Age(years)*					
< 40	58(24.3)	181(75.7)	1		
≥ 40	84(36.5)	146(63.5)	1.80	1.20–2.68	**0.003**
*Educational status*					
None/primary	12(60.0)	8(40.0)	5.51	2.05–14.81	**0.001**
Secondary	45(39.1)	70(60.9)	2.36	1.34–4.14	**0.003**
Tertiary	57(28.1)	146(71.9)	1.43	0.85–2.41	0.171
Postgraduate	28(21.4)	103(78.6)	1		
*Marital status*					
Single	31(23.0)	104(77.0)	1		
Married/cohabiting	95(31.7)	205(68.3)	1.55	0.97–2.49	0.065
Widowed/separated	16(47.1)	18(52.9)	2.98	1.36–6.53	**0.006**
*Employment status*					
Salary earner	53(26.6)	146(73.4)	0.75	0.46–1.22	0.239
Self-employment	49(33.1)	94(66.9)	1.01	0.61–1.69	0.955
Unemployed	40(32.8)	82(67.2)	1		
*Monthly income (Naira)*					
None	34(30.9)	76(69.1)	1		
< 50,000	48(34.3)	92(65.7)	1.17	0.68–1.99	0.573
50–100,000	24(30.4)	55(69.6)	0.98	0.52–1.83	0.938
> 100,000	36(25.7)	104(74.3)	0.77	0.44–1.35	0.364
*SM is cheaper*					
Yes	125(33.8)	245(66.2)	2.46	1.40–4.33	**0.002**
No	17(17.2)	82(82.8)	1		
*Correct definition of SM*					
Yes	125(30.3)	287(69.7)	1		
No	17(29.8)	40(70.2)	1.02	0.56–1.88	0.937
*SM is harmful*					
Yes	122(30.5)	278(69.5)	0.93	0.53–1.63	0.800
No	20(29.0)	49(71.0)	1		

*P* value < 0.05 are considered significant, SM self-medication, *binary logistic regression

After controlling for other variables in the multivariate logistic regression model, the predictors of self-medication against COVID-19 prevention and treatment were older age (aOR = 1.87, 95% CI: 1.11–3.13), no formal education (aOR = 3.78, 95% CI: 1.28–11.19), primary education (aOR = 2.15, 95% CI: 1.17–3.097) and the perception of self-medication as cheap (aOR = 2.29; 95CI: I.24–4.24) (Table 3).

**Table 3:** Predictors of self-medication against COVID-19 prevention among the respondents

Variable	aOR	95%CI	*P* value*
*Age(years)*			
< 40	1		
≥ 40	1.87	1.11–3.13	**0.018**
*Educational status*			
None/primary	3.78	1.28–11.19	**0.016**
Secondary	2.15	1.17–3.97	**0.014**
Tertiary	1.50	0.87–2.59	0.146
Postgraduate	1		
*Marital status*			
Single	1		
Married/cohabiting	1.31	0.74–2.33	0.352
Widow/separated	1.30	0.49–3.45	0.595
*SM is cheaper*			
Yes	2.29	1.24–4.24	**0.008**
No	1		

*P* value < 0.05 are considered significant, SM self-medication, *binary logistic regression

## Discussion

This study assessed the prevalence, pattern and predictors of self-medication for the prevention and or treatment of COVID-19 among residents in Umuahia, Abia State. One out of every three respondents practiced self-medication. The most commonly used drugs were herbal products mostly self-prepared. The predictors of self-medication were older age, low educational status and a perception of low cost for self-medication.

The prevalence of self-medication reported in this study was consistent with the findings of studies in Nigeria (41%) [30], Togo (34.2%) [31], Saudi Arabia (35.1%) [32] and Peru (33.9%) [33]. However, the prevalence of self-medication in this study was at variance with higher rates of 57%, 80.4%, 88.3% and 51.3% in Uganda, Jordan, Bangladesh and Peru, respectively [14, 34–36]. The lower rate observed in this study is related to the high level of awareness and knowledge of self-medication noted among the respondents, as more than three-quarter of the respondents were aware of self-medication, correctly defined self-medication and reported that self-medication is harmful. Furthermore, the heightened perceived fear of COVID-19 at the start of the pandemic has been significantly reduced with the introduction of vaccines. However, with rising AMR globally, there is a need to reduce self-medication to the barest minimum through the education and sensitization of residents on the dangers of self-medication and improved enforcement of OTC and POM drugs legislations.

In this study, the most frequently used drug formulation for self-medication was herbal products, and it was mostly self-prepared. This is similar to the results of studies conducted in Togo and Egypt where herbs and traditional medicines were most commonly used [31, 37]. This differs from the prevalent use of pain relievers [36] and antibiotics [5, 33], ivermectin [14, 38], vitamins and supplements [30, 31, 35] and antimalarials [30] as documented in other studies. In Nigeria, herbal treatments are usually patronized for the treatment of various ailments [39]. In this study, it was noted that family members and friends were the major sources of information for self-medication practices. This is consistent with a study done in Nigeria where it was noted that family and friends play a vital role in most people who use herbal medicines [39]. This is due in part to robust consumer awareness in mass media promoting its credibility and availability [40]. Many herbal products have been used to treat COVID-19 globally, but there is a need to provide safety monitoring data to guide its dosing and formulations [41, 42]. It is imperative that the stakeholders design public health enlightenment programmes on the hazards of herbal medicine especially for self-medication, to avert its potential adverse effects.

A majority of the respondents reported fear of isolation and stigmatization or discrimination as the triggers of self-medication. This is consistent with the findings of a population survey conducted in Nigeria [30]. It is known that stigmatization affects an individual’s overall well-being by reducing the health-seeking behaviour [43]. In the context of COVID-19, where early detection is paramount, this becomes a major challenge in the control of the pandemic. A major obstacle to compliance with isolation is concern about loss of income [44]. There is a need to use social influencers while communicating key messages during social mobilization exercises. Additionally, the creation of awareness should be done adequately without arousing fear.

In this study, age was statistically significantly associated with the uptake of self-medication for COVID-19. Older respondents (≥ 40 years) were more likely to self-medicate compared to the younger age groups (< 40 years). This finding agreed with other studies conducted in Nigeria, Serbia, Peru and China where higher odds of self-medication were observed among older persons [5, 45–47]. Conversely, this finding was at variance with studies done in Pakistan, India and Saudi Arabia [48–51]. The finding of this study can be explained by the fact that as people get older, they tend to be more intentional about their health in order to avoid the degenerative diseases associated with advance in age [52]. This means that they are more likely to be involved in polypharmacy with an increase in the occurrence of side effects. A study conducted in Nigeria has shown that older people are less hesitant to COVID-19 vaccine and as such will want to remain protected against the infection [53].

Self-medication was significantly associated with level of education as those without tertiary education were more likely to self-medicate compared to those who had attained tertiary education. This is similar to the findings of studies in Nigeria, Iran and Middle East [54–56]. However, several studies contrast this finding, showing that the highly educated are more likely to self-medicate [30, 31, 46, 48, 49, 51, 57, 58]. Less educated people are not optimally knowledgeable about their health, including the side effects of self-medication. Additionally, they are likely to be unemployed and may not have access to the National Health Insurance Scheme (NHIS), which makes it difficult for them to have full access to health care and they are likely to experience financial hardship in seeking health care services. There is a need for stakeholders to prioritize the role of education as a determinant of health.

Self-medication was associated with perceived cost of medication. Those who believed that self-medication was cheaper had higher odds of self-medication compared to their counterparts. As evidenced in studies [31, 59], cost of health services and other health service-related factors contribute to the likelihood of the populace to get involved in self-medication. Efforts should be made to increase universal health coverage to all, in line with the Sustainable Development Goal (SDG) goal 3. This will ensure equity needed to improve the health of the population and also reduce out of pocket expenditure (OOP).

The major strength of this study is that it is among the first studies in southeastern Nigeria to explore self-medication for COVID-19 prevention to the best of our searches. Another strength is that the general population was involved, in contrast to other studies involving only specified populations (pregnant women, civil servants) or institutionalized groups (university students, healthcare workers, in-or out-patients in hospitals). Few limitations were observed in this study. There was a possibility of selection bias as only those who had access to smart devices connected to the internet could participate in the study. This could affect the generalizability of the findings to the general populace. Use of self-reported data, as in this study, is prone to recall bias and social desirability bias. These were mitigated by attaching a showcard of possible drugs for easy identification and assuring the respondents of full confidentiality of their responses.

## Conclusion

Self-medication was prevalent among one-third of residents in Umuahia, Abia State. The most commonly used medication was herbal products mostly self-prepared. The major source of information for self-medication in this current study were family members and friends. Age, educational status and perception of cost significantly influenced self-medication practices for the prevention and treatment of COVID-19. We therefore, recommend that stakeholders conduct awareness and sensitization campaigns on the risks of self-medication targeting the older age groups and those with low educational status. Additionally, there is a need to create public enlightenment programmes on the hazards of herbal medicine use to avert the potential adverse effects (Additional files 1 and 2).

## Supplementary Information

**Additional file 1:** STROBE guideline checklist.

**Additional file 2:** Questionnaire.

## Data Availability

The datasets analyzed in the study are available from the corresponding author on reasonable request.

## Abbreviations

AMR: Antimicrobial resistance
AOR: Adjusted odds ratio
COR: Crude odds ratio
COVAX: COVID-19 Vaccines Global Access
IBM SPSS: Statistical Package for Social Sciences
NHIS: National Health Insurance Scheme
OOP: Out of pocket
OR: Odds ratio
OTC: Over-the-counter
POMs: Prescription-only medicines
SDGs: Sustainable development goals
SM: Self-medication
WHO: World Health Organization
