# Supplementary material for: Prevalence, pattern and predictors of self-medication for COVID-19 among residents in Umuahia, Abia State, Southeast Nigeria: policy and public health implications
Source: J Pharm Policy Pract. 2022 May 2;15:34. doi: 10.1186/s40545-022-00429-9 (PMC9058746; doi:10.1186/s40545-022-00429-9)
Supplement: Supplementary file 2 — Additional file 2: Questionnaire. [file 40545_2022_429_MOESM2_ESM.pdf]

QUESTIONNAIRE

Dear respondent,

We are researchers from the Department of Community Medicine Federal Medical Centre Umuahia researching the topic "Prevalence, pattern and predictors of self-medication for COVID-19 among residents in Umuahia, Abia State, Southeast Nigeria: Policy and public health implications". Please spare less than five minutes of your time to answer the questions below, on the subject matter. Your responses are completely anonymous and data collected will be well protected.

Your opinion, experiences, and responses on this subject are very important to us and will be treated as anonymous and your information will be handled confidentially. This will provide evidence as to why people resort to self-medication despite the availability of legislations against such. This survey is voluntary and you are free to opt out anytime you so desire. So, feel free to give us your frank and honest opinion.

In case of enquiries, please contact Dr. Amuzie Chidinma Ihuoma, 07038049596 ihuoma1712@yahoo.com, Department of Community Medicine, Umuahia Abia State.

* Required

Sociodemographic Information

Email address:

____

1. How old are you? (Age as at last birthday) *

____

2. Sex *

Mark only one oval.

◯ Female

◯ Male

3. Religion *

Mark only one oval.

◯ Christianity

◯ Islam

◯ African traditional religion

◯ Other:

4. If Christianity, what is your denomination?

Mark only one oval.

- ◯ Orthodox **(Anglican, Methodist, Presbyterian)**
- ◯ Pentecostal
- ◯ Catholic
- ◯ Other:

5. Educational Status *

Mark only one oval.

- ◯ None
- ◯ Primary
- ◯ Secondary
- ◯ Tertiary
- ◯ Post graduate
- ◯ Other:

6. Marital Status *

Mark only one oval.

- ◯ Single
- ◯ Married
- ◯ Cohabiting **(living together but not legally married)**
- ◯ Separated
- ◯ Widowed

7. Employment status *

Mark only one oval.

- ◯ Salary earner
- ◯ Self employed
- ◯ Unemployed
- ◯ Other:

8. Monthly household income (Naira) *

Mark only one oval.

- ◯ <50,000
- ◯ 50,000-100,000
- ◯ >100,000
- ◯ No income

Awareness/knowledge on self-medication

9. Have you ever heard about self-medication? *

Mark only one oval.

- Yes
- No
- I don't know

10. What is self-medication?*

Mark only one oval.

- Buying drugs prescribed by the doctor from chemist shop instead of hospital pharmacy
- Taking medication or herbal treatment without prescription from a qualified doctor after a proper consultation
- Taking prescribed drugs at home instead of taking them at hospital where doctor can see you)
- I don't know

11. Self-medication is harmful? *

Mark only one oval.

- Yes
- No
- I don't know

12. Self-medication is cheaper than seeking medical services? *

Mark only one oval.

- Yes
- No
- I don't know

Proportion of self-medication for COVID-19

13. In the past 3 months have you taken any medication or herbal treatment to prevent COVID-19 without prescription from doctor? *

Mark only one oval.

- Yes *Skip to question 14*
- No *Skip to question 17*

Self-Medication practices

14. Which drugs/supplement did you use for the COVID-19 prevention/treatment ?*

Check all that apply

- [ ] Azithromycin
- [ ] Vitamin supplements
- [ ] Ivermectin
- [ ] Calcium supplement
- [ ] Herbal products
- [ ] Ciprofloxacin
- [ ] Amoxillin/clavulanic acid (Augmentin)
- [ ] Erythromycin
- [ ] Pain relievers (Paracetamol, ibuprofen, etc)
- [ ] Hydroxychloroquine/chloroquine
- [ ] Other antimalarials
- [ ] Other: ______________________

15. Where did you get your source of information for acquiring the medications? *

Check all that apply

- [ ] Internet
- [ ] Friends
- [ ] Colleagues
- [ ] Family members
- [ ] Self
- [ ] Doctor
- [ ] Other _________________________________

16. Where did you get the medication or herbal treatment from? *

Check all that apply.

- [ ] Patent medical vendors (Unregistered drug stores)
- [ ] Pharmacy stores (Registered drug stores)
- [ ] Leftovers (remnants of previous medication used by self/ family member(s) I prepared it myself

Other: [ ] ____________________________________

Triggers of self-medication

17. The fear of being infected through a known/confirmed case of COVID-19 *

Mark only one oval.

- Yes
- No
- Maybe

18. The fear of being isolated due to COVID-19 *

Mark only one oval.

- Yes
- No
- Maybe

19. The fear of being stigmatized or discriminated due to COVID-19 *

Mark only one oval.

- Yes
- No
- Maybe

20. The anticipated delay in getting tested and retrieving COVID-19 results *

Mark only one oval.

- Yes
- No
- Maybe

21. Anticipated poor health services in the COVID-19 isolation areas *

Mark only one oval.

- Yes
- No
- Maybe

22. Heard about the death of a colleague, friend or an acquaintance from COVID-19*

Mark only one oval.

- Yes
- No
- Maybe

23. Being influenced by friends to use self-medication to COVID-19 prevention or treatment *

Mark only one oval.

- Yes
- No
- Maybe

24. Being influenced by the mass media/social media to use self-medication to COVID-19 prevention or treatment *

Mark only one oval.

- Yes
- No
- Maybe

Thank you for your time and cooperation

End of survey
